# Vitamin K2 and cotylenin A synergistically induce monocytic differentiation and growth arrest along with the suppression of *c-MYC* expression and induction of cyclin G2 expression in human leukemia HL-60 cells

**DOI:** 10.3892/ijo.2015.3028

**Published:** 2015-06-04

**Authors:** YASUHISA MANIWA, TAKASHI KASUKABE, SHUNICHI KUMAKURA

**Affiliations:** 1 Masuda Red Cross Hospital, Masuda 698-8501, Japan; 2 Department of Medical Education and Research, Faculty of Medicine, Shimane University, Izumo 693-8501, Japan

**Keywords:** vitamin K2, cotylenin A, differentiation, growth arrest, acute myeloid leukemia cells, *c-MYC*, cyclin G2

## Abstract

Although all-*trans* retinoic acid (ATRA) is a standard and effective drug used for differentiation therapy in acute promyelocytic leukemia, ATRA-resistant leukemia cells ultimately emerge during this treatment. Therefore, the development of new drugs or effective combination therapy is urgently needed. We demonstrate that the combined treatment of vitamin K2 and cotylenin A synergistically induced monocytic differentiation in HL-60 cells. This combined treatment also synergistically induced NBT-reducing activity and non-specific esterase-positive cells as well as morphological changes to monocyte/macrophage-like cells. Vitamin K2 and cotylenin A cooperatively inhibited the proliferation of HL-60 cells in short-term and long-term cultures. This treatment also induced growth arrest at the G1 phase. Although 5 μg/ml cotylenin A or 5 μM vitamin K2 alone reduced *c-MYC* gene expression in HL-60 cells to approximately 45% or 80% that of control cells, respectively, the combined treatment almost completely suppressed *c-MYC* gene expression. We also demonstrated that the combined treatment of vitamin K2 and cotylenin A synergistically induced the expression of cyclin G2, which had a positive effect on the promotion and maintenance of cell cycle arrest. These results suggest that the combination of vitamin K2 and cotylenin A has therapeutic value in the treatment of acute myeloid leukemia.

## Introduction

Acute myeloid leukemia (AML) is the most common type of leukemia in adults, and occurs in approximately one-third of newly diagnosed patients, and remains one of the most difficult hematological malignancies to treat (the 5-year overall survival rate is 20–30% for adult primary AML) (1,2). AML is characterized by the proliferation of clonal precursor myeloid cells with arrested differentiation (3). In contrast to the poor prognosis of most patients with AML, the use of differentiation therapy with all-*trans*-retinoic acid (ATRA) for acute promyelocytic leukemia (APL), a distinct type of AML, has revolutionized therapy for this disease by converting it from fatal to curable (4). However, ATRA is not effective in other AMLs. Furthermore, many APL patients treated with ATRA fail to respond or invariably relapse. Therefore, alternative or combination therapies are needed to improve the prognosis and survival of patients.

Cotylenin A (CN-A), which is a fucicoccan-diterpene glycoside with a complex sugar moiety, was originally isolated as a plant growth regulator and has been shown to affect several physiological processes in higher plants (5). We previously reported that CN-A exhibited potent differentiation-inducing activity in several human and murine myeloid leukemia cell lines and in leukemia cells that were freshly isolated from patients with AML (6–9). Furthermore, the administration of CN-A significantly prolonged the survival of mice with severe combined immunodeficiency that had been inoculated with the APL cells of the NB-4 cell line (10).

Previous studies reported that vitamin K2 (VK2) effectively induced apoptosis in various types of primary cultured leukemia cells and leukemia cell lines *in vitro* (11–13) as well as in solid tumor cells (14–16). On the other hand, in contrast to the induction of apoptosis in leukemia cells, VK2 has been shown to exhibit differentiation-inducing activity in AML cell lines, such as HL-60 and U937, *in vitro* (17–19). Sada *et al* found that VK2 also had differentiation-promoting effects on myeloid lineage progenitors (20).

Since *c-MYC* is aberrantly expressed in a wide variety of human solid tumors (21) as well as in leukemia (22), it is an attractive target for cancer therapy. The downregulation of *c-MYC* is known to play a crucial role in ATRA-induced growth arrest and myeloid differentiation of AML (23–27). In addition, previous findings, including ours, indicated that the expression of cyclin G2 was significantly upregulated during cell cycle arrest responses to diverse growth-inhibitory signals and strongly repressed by mitogens, suggesting the positive role of cyclin G2 in the promotion or maintenance of cell cycle arrest (28–30). In order to identify useful new differentiation inducers and effective combination treatments for various types of AML and APL, we searched for substances capable of inducing cell differentiation and the expression of cyclin G2 as well as strongly suppressing the expression of *c-MYC* in HL-60 cells. In the present study, we demonstrated that the combined treatment of VK2 and CN-A synergistically induced monocytic differentiation in HL-60 cells and cooperatively inhibited cell proliferation showing G1 arrest. Furthermore, we showed that the combined treatment of VK2 and CN-A efficiently suppressed the expression of *c-MYC* and cooperatively induced the expression of cyclin G2.

## Materials and methods

### Reagents

VK2, nitroblue tetrazolium (NBT), all-*trans* retinoic acid (ATRA), 1α,25-dihydroxyvitamin D3 (VD3), and 12-*O*-tetradecanoylphorbol-13-acetate (TPA) were purchased from Sigma-Aldrich Inc. (St. Louis, MO, USA). CN-A was purified from a stock ethyl acetate extract obtained from the culture filtrate of *Cladosporium* fungus sp. 501-7 W by flash chromatography on a silica gel with >99% purity (5).

### Cells and cell culture

Human AML HL-60 cells were cultured in RPMI-1640 medium (Sigma-Aldrich Inc.) supplemented with 10% heat-inactivated fetal bovine serum and 80 μg/ml gentamicin sulfate (MSD K.K, Tokyo, Japan) at 37°C in a humidified atmosphere of 5% CO_2_ in air.

### Assay of cell growth

Cells were plated in multidishes (Falcon, Corning Inc., Corning, NY, USA) at a density of 2.5×10^4^ cells/ml and incubated with or without the test compounds. Cell numbers were counted with a model Z1 Coulter Counter (Beckman Coulter Inc., Miami, FL, USA).

### NBT reduction assay

The reduction of NBT was assayed colorimetrically as previously described (31). Briefly, cells were incubated in 1 ml of serum-free medium containing 1 mg/ml NBT and 100 ng/ml TPA at 37°C for 60 min. The reaction was stopped by adding HCl. Formazan solution at 560 nm was measured in a spectrophotometer (DU730, Beckman Coulter Inc.).

### Assessment of monocytic differentiation

In order to assess monocytic differentiation, non-specific esterase staining was performed using an Esterase Staining kit (Muto Chemical Co., Tokyo, Japan).

### Assessment of morphological differentiation

Morphological changes were examined in cell smears using light microscopy of cytospin preparations stained with May-Grunwald-Giemsa solution (Merck, Darmstadt, Germany).

### Cell cycle analysis

Cells were plated in 60-mm plastic dishes at a density of 1×10^5^ cells/ml and incubated with VK2 in the absence or presence of CN-A. After 96 h, the cells were washed with phosphate-buffered saline (PBS) and fixed gently in 100% ethanol at 4°C for 30 min. Cells were suspended in propidium iodide (PI)-RNase solution, which contained 50 μg/ml PI (MBL Co. Ltd., Nagoya, Japan) and 0.1 mg/ml RNase (Sigma-Aldrich Inc.) in PBS for 30 min at room temperature. The cell cycle analysis was performed by flow cytometry (BD FACSCalibur, Becton Dickinson, East Rutherford, NJ, USA).

### RNA extraction and determination of mRNA levels by reverse transcriptase (RT)-quantitative polymerase chain reaction (qPCR)

RNA was extracted using an RNeasy Plus Mini kit (Qiagen, Valencia, CA, USA) according to the manufacturer’s instructions. Total RNA (1 μg) from leukemia cells was reverse transcribed with the ReverTra Ace qPCR RT kit (Toyobo Co. Ltd., Osaka, Japan). qPCR using the SYBER Green method was carried out with the Thunderbird SYBER qPCR Mix (Toyobo Co.) on a Thermal Cycler Dice Real-time PCR instrument (Takara Bio, Shiga, Japan) according to the manufacturer’s instructions. Real-time PCR results were calculated according to the following protocol: Relative expression level=2^−ΔCt^, where ΔCt=Ct (gene of interest) - Ct (housekeeping gene). The *c-MYC* primers used for qPCR were: forward, 5′-TTCGGGT AGTGGAAAACCAG-3′ and reverse, 5′-CAGCAGCTCGAA TTTCTTCC-3′. The *GAPDH* primers used for qPCR were: forward, 5′-GACGCTGGGGCTGGCATTG-3′ and reverse, 5′-GCTGGTGGTCCAGGGGTC-3′ (32). The cyclin G2 primers used for qPCR were: forward, 5′-ATCGTTTCAAG GCGCACAG-3′ and reverse, 5′-CAACCCCCCTCAGGTA TCG-3′ (33). The P21/CIP1 primers used for qPCR were: forward, 5′-CGATGCCAACCTCCTCAACGA-3′ and reverse, 5′-TCGCAGACCTCCAGCATCCA-3 (34).

## Results

### Effects of the combined treatment of vitamin K2 (VK2) and cotylenin A (CN-A) on the cell proliferation of HL-60 cells

**Figure 1:**
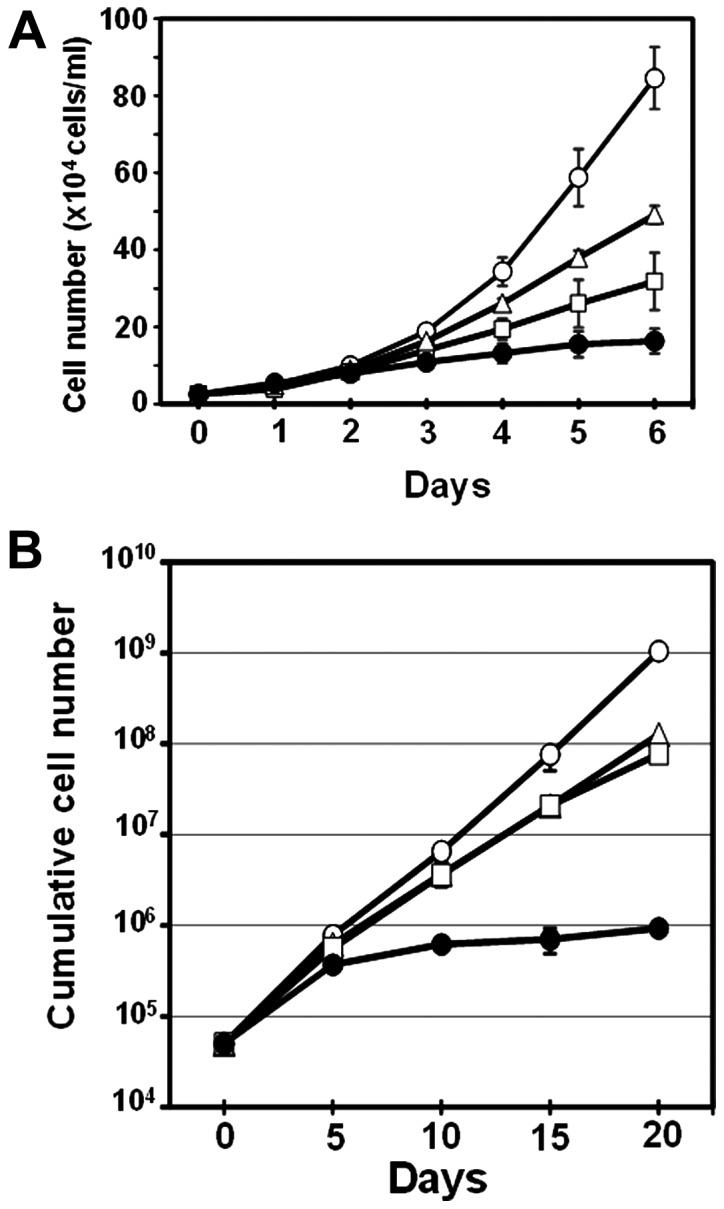
Effects of the combined treatment of vitamin K2 (VK2) and cotylenin A (CN-A) on cell proliferation in HL-60 cells. (A) HL-60 cells (2.5×10^4^ cells/ml) were cultured without (open circle) or with 5 μM VK2 (open square), 5 μg/ml CN-A (open triangle), or 5 μM VK2 plus 5 μg/ml CN-A (closed circle) for the indicated number of days. (B) HL-60 cells (5×10^4^ cells/ml) were cultured without (open circle) or with 10 μM VK2 (open square), 5 μg/ml CN-A (open triangle), or 10 μM VK2 plus 5 μg/ml CN-A (closed circle) for the indicated number of days. The culture medium was replaced by fresh medium once every 5 days. Cell density was maintained at 5–90×10^4^ cells/ml. Values are expressed as the mean ± SD of three determinations.

HL-60 cells (2.5×10^4^ cells/ml) were cultured without or with VK2, CN-A, or VK2 plus CN-A for 6 days. Fig. 1A shows the time course of the combined effects of VK2 and CN-A on cell growth. The growth of HL-60 cells was moderately inhibited by VK2 (5 μM) or CN-A (5 μg/ml) alone, but was still observed until at least 6 days; however, no significant changes were observed in the cell number after 4 days of the treatment with the combination of both VK2 and CN-A (Fig. 1A). We also examined the long-term effects of the combined treatment of VK2 and CN-A on the proliferation of HL-60 cells. HL-60 cells (5×10^4^ cells/ml) were cultured without or with 10 μM VK2, 5 μg/ml CN-A, or 10 μM VK2 plus 5 μg/ml CN-A for 20 days (Fig. 1B). The culture medium was replaced by fresh medium once every 5 days. Although the growth rate of VK2- or CN-A-treated cells was significantly lower than that of control cells under these culture conditions, the cell number markedly increased (100-fold between days 5 and 20). On the other hand, cell growth was greatly inhibited by the combined treatment of VK2 and CN-A, and the cell number was almost the same as that at day 5 (Fig. 1B).

### VK2 and CN-A synergistically induced monocytic differentiation in HL-60 cells

**Figure 2:**
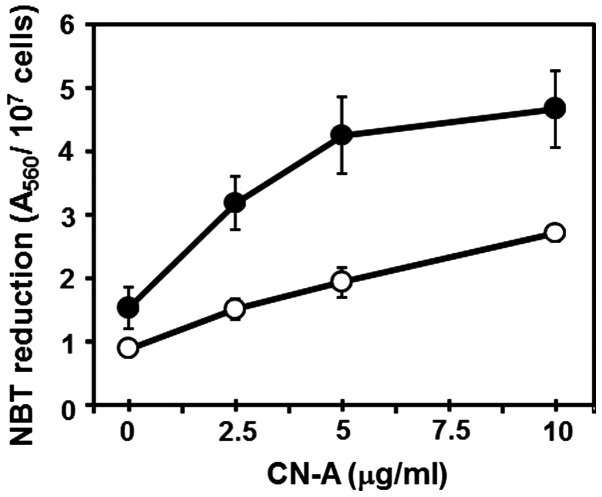
Induction of NBT reduction in HL-60 cells by the treatment with CN-A and VK2. HL-60 cells (2.5×10^4^ cells/ml) were cultured with CN-A in the presence (closed circle) and absence (open circle) of 10 μM VK2 for 6 days. NBT-reducing activities were determined. Values are expressed as the mean ± SD of three determinations.

**Figure 3:**
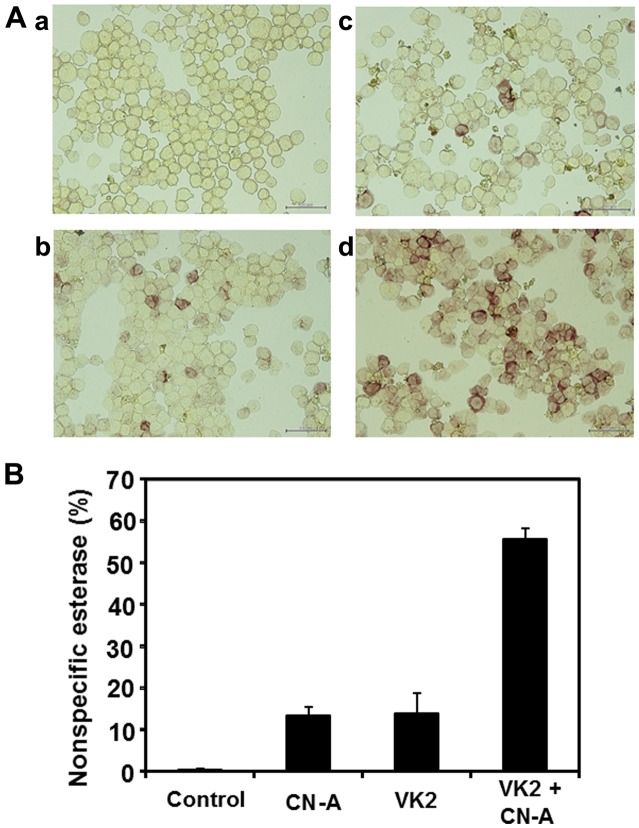
Induction of non-specific esterase in HL-60 cells by the treatment with CN-A and VK2. (A) HL-60 cells (2.5×10^4^ cells/ml) were cultured without (a) or with 10 μg/ml CN-A (b), 10 μM VK2 (c), or 10 μM VK2 plus 10 μg/ml CN-A (d) for 5 days. The results are representative of 3 independent experiments. (B) Non-specific esterase-positive cells were counted by microscopy. Data are representative of the mean ± SD of three determinations.

**Figure 4:**
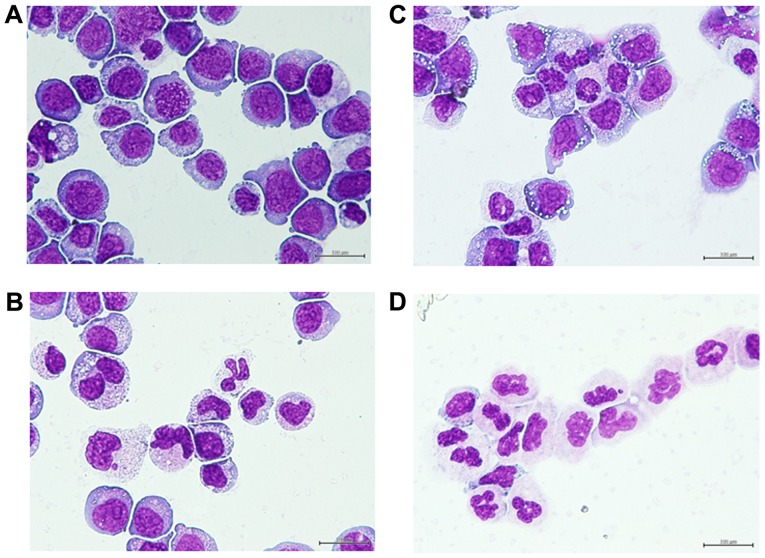
Induction of morphological differentiation in HL-60 cells by the treatment with CN-A and VK2. HL-60 cells (2.5×10^4^ cells/ml) were cultured without (A) or with 5 μg/ml CN-A (B), 5 μM VK2 (C), or 5 μM VK2 plus 5 μg/ml CN-A (D) for 6 days. The results are representative of 3 independent experiments.

We examined the combined effects of VK2 and CN-A on the induction of differentiation of HL-60 cells because VK2 or CN-A alone are inducers of differentiation in HL-60 cells (6,7,17,18). HL-60 cells (2.5×10^4^ cells/ml) were cultured with CN-A in the presence or absence of VK2 for 6 days. CN-A and 10 μM VK2 synergistically induced the reduction of NBT (one of the typical myelo/monocytic differentiation markers of human leukemia cells) (Fig. 2). We then determined whether the induction of differentiation induced with VK2 plus CN-A was a granulocytic or monocytic lineage. HL-60 cells (2.5×10^4^ cells/ml) were cultured without or with 10 μg/ml CN-A, 10 μM VK2, or 10 μM VK2 plus 10 μg/ml CN-A for 5 days (Fig. 3A). Nonspecific esterase-positive cells were counted under a microscope (Fig. 3B). Cells treated with CN-A plus VK2 synergistically became positive for nonspecific esterase (Fig. 3A-d and B), whereas those treated with CN-A or VK2 alone became weakly positive (Fig. 3A–b, A–c and B). The combined treatment of VK2 and CN-A also induced the marked morphological differentiation of HL-60 cells (Fig. 4D), whereas VK2 or CN-A alone induced the intermediate stage of differentiation (Fig. 4B and C). These results indicated that the treatment of HL-60 cells with VK2 and CN-A effectively induced monocytic differentiation.

### Induction of G1 arrest in HL-60 cells with VK2 plus CN-A

**Figure 5:**
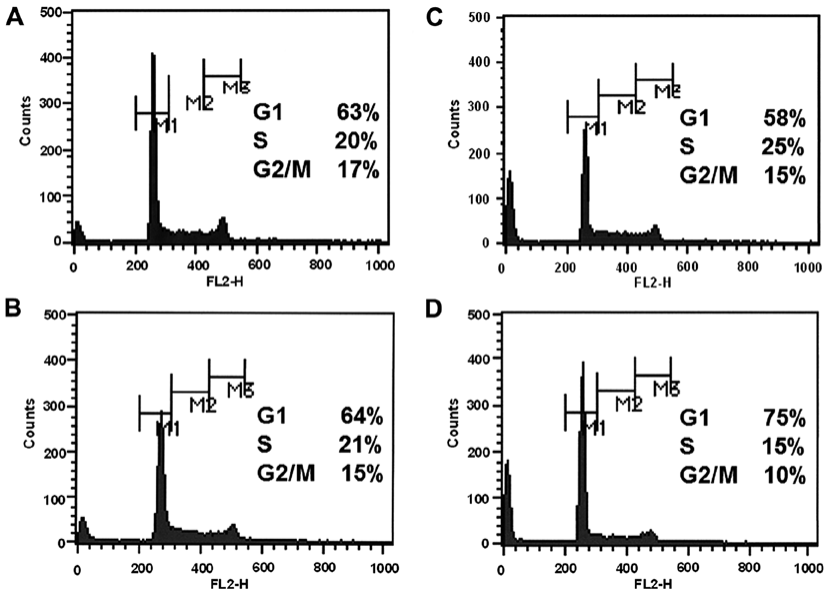
Induction of G1 arrest in HL-60 cells with VK2 plus CN-A. HL-60 cells (1×10^5^ cells/ml) were cultured without (A) or with 10 μg/ml CN-A (B), 10 μM VK2 (C), or 10 μM VK2 plus 10 μg/ml CN-A (D) for 4 days and a cell cycle analysis was performed using flow cytometry. Ten thousand cells were scored in each cytoplasmic profile. The results are representative of 3 independent experiments.

In order to more clearly understand the combined effects of VK2 and CN-A on cell growth, we exposed HL-60 cells (1×10^5^ cells/ml) to 10 μM VK2 plus 10 μg/ml CN-A, and then measured changes in cell cycle distribution after 4 days (Fig. 5). Under these culture conditions, VK2 or CN-A alone did not markedly affect the cell cycle (Fig. 5B and C). On the other hand, the percentage of cells in the G1 phase was significantly increased from 63% to 75% (Fig. 5A and D). The percentages of cells in the S phase and G2/M phase were inversely decreased in response to the combined treatment of VK2 and CN-A (Fig. 5A and D).

### Combined treatment of VK2 and CN-A synergistically inhibited c-MYC gene expression in HL-60 cells

**Figure 6:**
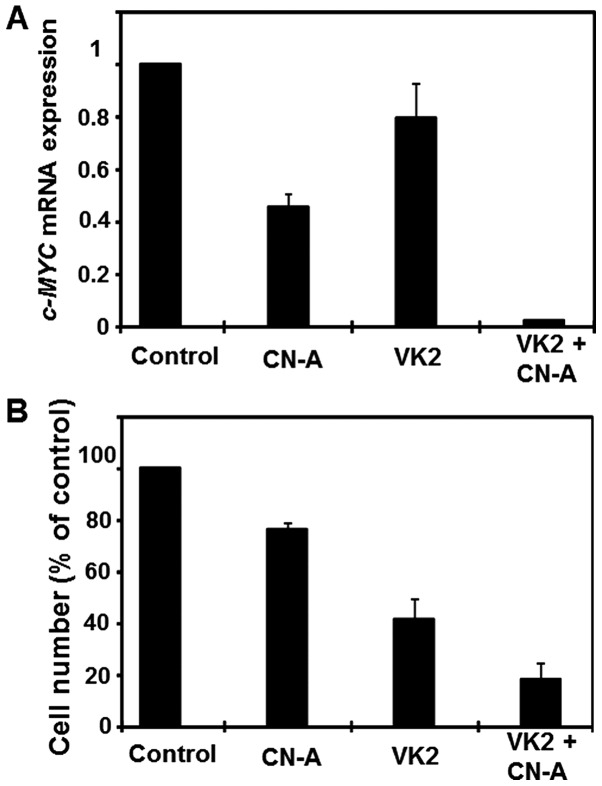
The combined treatment of CN-A and VK2 synergistically inhibited *c-MYC* gene expression in HL-60 cells. HL-60 cells (2.5×10^4^ cells/ml) were cultured without or with 5 μg/ml CN-A, 5 μM VK2, or 5 μM VK2 plus 5 μg/ml CN-A for 6 days. c-MYC gene expression (A) was examined by RT-qPCR and the number of cells (B) was measured using a cell counter. Data are representative of the mean ± SD of three determinations.

Previous studies reported that the induction of differentiation and growth arrest in HL-60 cells was associated with the suppression of *c-MYC* gene expression (23–27); therefore, we investigated whether the combined treatment of CN-A and VK2 synergistically inhibited *c-MYC* gene expression in HL-60 cells. HL-60 cells (2.5×10^4^ cells/ml) were cultured without or with VK2 plus CN-A for 6 days. Although 5 μg/ml CN-A or 5 μM VK2 alone inhibited *c-MYC* gene expression in HL-60 cells to approximately 45 or 80% that of control cells, respectively, the combined treatment almost completely suppressed *c-MYC* gene expression (>95% inhibition) (Fig. 6A). This synergistic inhibition of *c-MYC* gene expression in HL-60 cells was also observed when HL-60 cells were treated with CN-A and VK2 for 4 days (data not shown). As described above, the combined treatment of CN-A and VK2 more strongly inhibited cell growth than that of CN-A or VK2 alone (Fig. 6B) and clearly induced monocytic differentiation (Fig. 4).

### Combined treatment of VK2 and CN-A synergistically induced cyclin G2 gene expression in HL-60 cells

**Figure 7:**
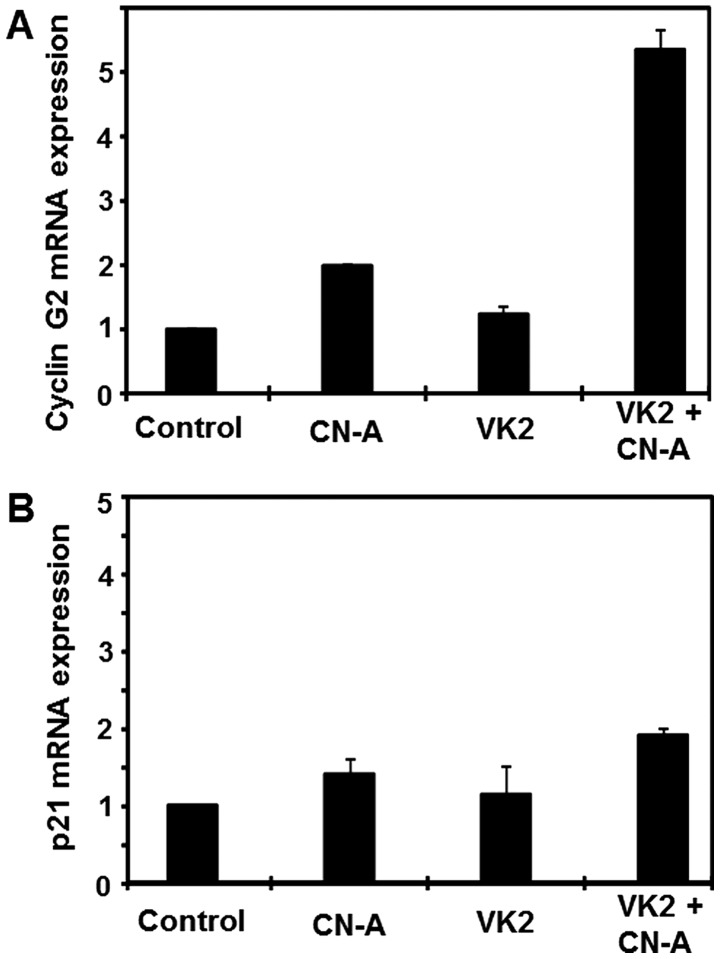
The combined treatment of CN-A and VK2 markedly induced cyclin G2 gene expression in HL-60 cells. HL-60 cells (2.5×10^4^ cells/ml) were cultured without or with 5 μg/ml CN-A, 5 μM VK2, or 5 μM VK2 plus 5 μg/ml CN-A for 6 days. Cyclin G2 (A) and p21/CIP1 gene expression (B) was examined by RT-qPCR. Data are representative of the mean ± SD of three determinations.

Previous findings, including ours, indicated that the expression of cyclin G2 was significantly upregulated during cell cycle arrest responses to diverse growth-inhibitory signals and strongly repressed by mitogens, suggesting a positive role for cyclin G2 in the promotion or maintenance of cell cycle arrest (28–30). Therefore, we determined whether the differentiation of HL-60 cells induced with VK2 and CN-A was accompanied by the induction of cyclin G2 expression. Cyclin G2 gene expression was markedly induced (>5-fold) in VK2 plus CN-A-treated HL-60 cells (Fig. 7A). The expression of cyclin G2 was approximately 2-fold higher in CN-A-treated HL-60 cells than in control cells, whereas VK2-treated cells showed only a marginal increase (Fig. 7A). Similar results were obtained when HL-60 cells were treated with VK2 plus CN-A for 4 days (data not shown).

We also examined the gene expression levels of several cell cycle regulators such as p21/CIP1, p27/KIP1, and cyclin D1. We did not observe the marked induction (>2-fold) of the expression of p21/CIP1 (Fig. 7B), p27/KIP1 (data not shown), or cyclin D1 (data not shown) in VK2-, CN-A-, or VK2 plus CN-A-treated HL-60 cells.

### Effects of VK2 on the expression of c-MYC and cyclin G2 in VD3- or ATRA-treated HL-60 cells

**Figure 8:**
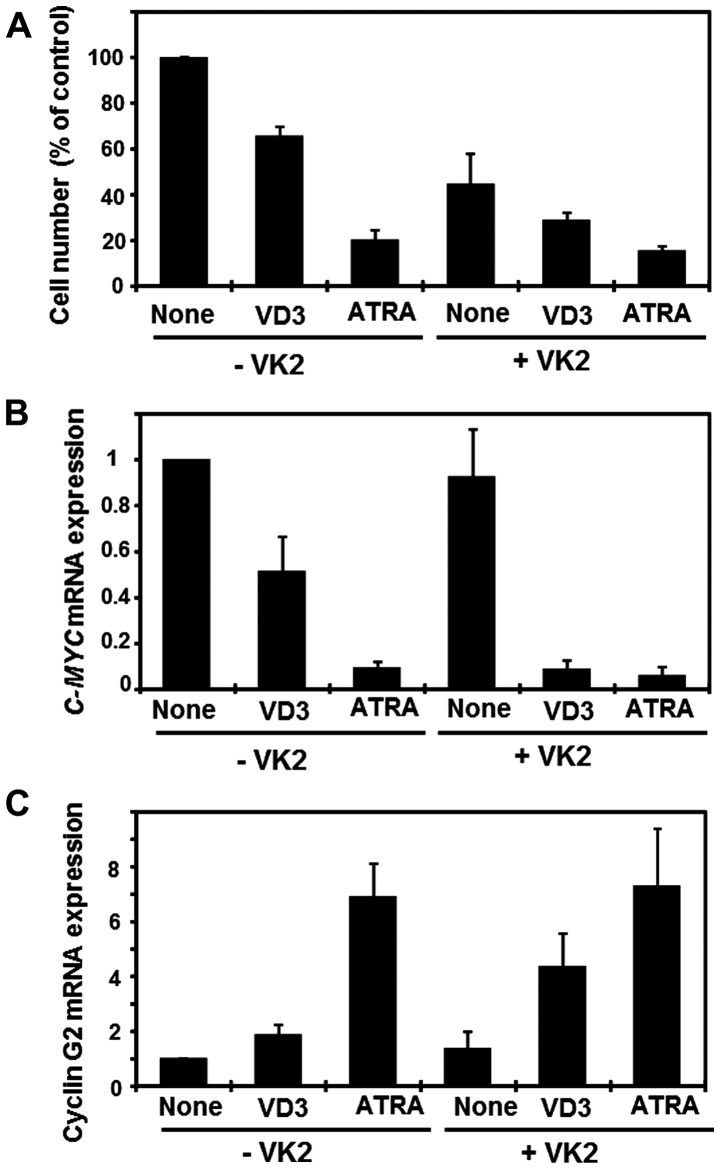
Effects of VK2 on cell growth, *c-MYC* expression, and cyclin G2 expression in HL-60 cells in the presence of VD3 or ATRA. HL-60 cells (2.5×10^4^ cells/ml) were cultured without or with 100 nM VD3 or 75 nM ATRA in the presence or absence of 5 μM VK2 for 6 days. Cell number (A) and the expression of *c-MYC* (B) and cyclin G2 (C) were determined. Data are representative of the mean ± SD of three determinations.

We examined the effects of VK2 on cell growth and the expression of *c-MYC* and cyclin G2 in HL-60 cells treated with two typical differentiation inducers. VD3 is one of the most potent monocytic differentiation inducers identified to date (35,36). Although VD3 or VK2 alone inhibited cell growth to approximately 65 or 45% that of control cells and also suppressed the expression of *c-MYC* to approximately 50 or 90% that of control cells, respectively, the combined treatment of VD3 and VK2 inhibited cell growth to approximately 30% that of control cells and suppressed the expression of *c-MYC* to <10% that of control cells (Fig. 8A and B). Furthermore, VD3 plus VK2 cooperatively induced cyclin G2 gene expression more than that of the additive manner (Fig. 8C). On the other hand, ATRA alone, which is a standard drug used for differentiation therapy in APL, inhibited cell growth to approximately 20% that of control cells, suppressed *c-MYC* expression to <10% that of control cells, and markedly induced cyclin G2 gene expression (6.9-fold) (Fig. 8). Under this treatment condition using ATRA, VK2 marginally increased the effects of ATRA on cell growth, *c-MYC* expression, and cyclin G2 expression (Fig. 8).

## Discussion

Vitamin Ks (VK) are known to act as co-factor for the γ-carboxylation of prothrombin and other VK-dependent coagulation factors (37). VK promotes osteogenesis through the γ-carboxylation of glutamate residues in osteocalcin. VK2 is a naturally-occurring and the main form of vitamin K in the tissues. A synthetic VK2 analog has been approved as an anti-osteoporotic medicine by the Ministry of Health, Labor and Welfare in Japan. The safety of the long-term administration of VK2 has been well established (38). Although the exact mechanism has not yet been elucidated in detail, VK2 and their analogs have been shown to inhibit the survival of various cancer cell lines (14–16) and leukemia cells (11–13). Furthermore, previous studies reported that VK2 exhibited some differentiation-inducing activity in AML cell lines *in vitro* (17–19). Only VK2 alone induced the intermediate stage of differentiation in HL-60 cells in the present study (Fig. 4D) and, even at higher concentrations (>10 μM), VK2 could not induce mature differentiation, but induced apoptosis (data not shown). As VK2 is a naturally-occurring, safe, and clinically-utilized agent, we searched for substances that could enhance the differentiation-inducing activity of VK2. We found that CN-A, a differentiation inducer, synergistically induced the differentiation of HL-60 cells along with growth arrest, and markedly suppressed the expression of *c-MYC* and induction of cyclin G2 expression. This is the first study to examine the effects of VK2 plus CN-A on the induction of differentiation and expression of growth arrest-associated genes such as *c-MYC* and cyclin G2.

The proto-oncogene *c-MYC* has been shown to play an important role in cellular metabolism, apoptosis, differentiation, cell cycle progress and tumorigenesis (36–43). The expression of *c-MYC* in particular was found to contribute to leukemogenesis and promote the progression of leukemia (43). The downregulation of *c-MYC* is critical for the ATRA-induced growth arrest and myeloid differentiation of AML (23–27). The inhibition of *c-MYC* was also shown to suppress the proliferation and induce the differentiation of primary AML cells (27). Furthermore, the overexpression of *c-MYC* in an APL cell line inversely inhibited ATRA-induced cell differentiation (27). These findings indicate that *c-MYC* is an attractive target for differentiation therapy.

In the present study, we found that VK2 markedly enhanced the downregulation of *c-MYC* gene expression induced by differentiation inducers, whereas VK2 alone at the doses used weakly suppressed gene expression (Figs. 6A and 8B). The combined treatment of VK2 and CN-A exhibited the most potent suppressive effects on *c-MYC* gene expression among the inducers or their combinations tested. This combined treatment reduced the expression of *c-MYC* to approximately one fortieth that of control levels (Fig. 6A), and synergistically induced differentiation (Figs. 3 and 4) and growth arrest (Figs. 1 and 5). Although VK2 also effectively enhanced the suppressive effects of *c-MYC* expression induced by VD3 (Fig. 8B), VK2 plus VD3 reduced the expression of *c-MYC* less than that of VK2 plus CN-A (Figs. 6A and 8B). Furthermore, no derivative of active vitamin D3 has so far been used clinically as an anticancer agent because of the side effect of hypercalcemia (44). These results suggest that the combination of VK2 and CN-A has therapeutic value in the treatment of AML. Furthermore, since we previously found that CN-A was also capable of stimulating the functional and morphological differentiation of ATRA-resistant APL cells (10), the combined treatment of VK2 plus CN-A may be useful for differentiation therapy in retinoid-resistant leukemia.

The expression of cyclin G2 was shown to be significantly upregulated during cell cycle arrest responses to diverse growth-inhibitory signals and strongly repressed by mitogens, suggesting the positive role of cyclin G2 in the promotion or maintenance of cell cycle arrest (28–30). We previously reported that the combination of differentiation inducers including CN-A effectively inhibited the proliferation of several human breast cancer cell lines as well as leukemia cells (27,43). This treatment induced growth arrest in cells at the G1 phase rather than apoptosis and also rapidly and markedly induced cyclin G2 gene expression (29,45).

Cyclin G2 knockdown induced by cyclin G2 small interfering RNA markedly reduced the potency of CN-A plus other inducers to induce growth inhibition. Ectopically inducible cyclin G2 expression was shown to potently inhibit the proliferation of breast cancer cells (29). Therefore, for more effective differentiation therapy in AML, it is necessary to find new differentiation inducers or combination therapies that can induce cell differentiation and growth arrest. We have searched for substances that are capable of inducing cell differentiation and the expression of cyclin G2, and that can also strongly suppress the expression of *c-MYC* in HL-60 cells. We showed that the treatment with VK2 plus CN-A induced functional and morphological differentiation as well as growth arrest in HL-60 AML cells. Furthermore, this treatment almost completely suppressed the expression of *c-MYC* and markedly induced the expression of cyclin G2. Therefore, our results suggest an attractive combination for effective differentiation therapy in human myeloid leukemia. More detailed studies on the mechanisms underlying this effective combined treatment of VK2 plus CN-A are required.
